# Supplementary material for: Investigating clinic transfers among HIV patients considered lost to follow-up to improve understanding of the HIV care cascade: Findings from a cohort study in rural north-eastern South Africa
Source: PLOS Glob Public Health. 2022 May 24;2(5):e0000296. doi: 10.1371/journal.pgph.0000296 (PMC10022370; doi:10.1371/journal.pgph.0000296)
Supplement: Attachment — Submitted filename: Reviewer comments_PLOS_new.docx [file pgph.0000296.s002.docx]

February 17, 2022

Ruwan Ratnayake

Academic Editor

PLOS Global Public Health

RE: Re-submission of the Manuscript PGPH-D-21-00970

Dear Editor,

Thank you very much for reviewing our manuscript. We greatly appreciate the editor’s and reviewers’ comments and suggestions. We have taken these into account and revised the manuscript accordingly.

We have attached a manuscript with the changes highlighted as well as a clean version.

Please find attached a point-by-point response to the reviewers’ concerns. We hope that you find these responses satisfactory and look forward to hearing back from you soon.

Sincerely,

Dr David Etoori

(On behalf of the authors)

**Editor:**

**Thank you for sharing your important work with PLOS Global Public Health. You will find comments from two reviewers attached. In general, please pay close attention to, and respond completely to, each of their comments. I have four additional comments:**

**1. Please pay particular attention to the comments from reviewer 2 that focuses on overlapping text with other publications. We noticed minor occurrences of overlapping text with the following publication: Clouse K, Vermund SH, Maskew M, Lurie MN, MacLeod 362 W, Malete G, et al. Mobility and Clinic Switching Among Postpartum Women Considered Lost to HIV Care in South Africa. J Acquir Immune Defic Syndr 1999. 2017 01;74(4):383–9. Please check all text for similarities with this, and other, publications. Ensure that you cite all your sources (including your own works), and quote or rephrase any duplicated text outside the methods section. Further consideration is dependent on these concerns being addressed.**

We apologise for this genuine oversight. Between drafts, it would appear a couple of references were lost. We have now rephrased or deleted statements that are similar to this publication and have also added citations to credit the authors (lines 172-176, 308-310, 332-333).

**2. Describe each figure and table in the text. For example, the circle plots are not fully described and therefore, it is difficult to understand the content, and how they fit into the findings.**

Thank you for this suggestion. We have now described the Figures within the text (lines 211-214, 220-222, 235-238). We have further clarified how to interpret Figures 2 and 4 (lines 625-638)

**3. Please provide some insight on the importance of your findings on LTFU and methodologies (using health and demographic surveillance tools to trace patients) to global health practice in general (and subsequently, this journal). This is an important opportunity as the readership of PLOS Global Public Health is not limited to HIV/AIDS practitioners and researchers. I note that the process of tracing patients is well described in the methods, but the discussion needs more work in terms of describing the important of the outcomes and transferability of these methods elsewhere.**

Thank you for this suggestion. We have now added some more insights into our approach and the transferability of our methods (lines 337-350).

*“The use of linked demographic surveillance and clinic data is a novel approach in this setting and our findings show that it has some promise. This approach allowed us to utilise unique HDSS identifiers to follow patients between health facilities with 43% of undocumented transfers identified through this linkage. Our approach is reliant on an accurate linkage algorithm and given that some clinic records did not match to an HDSS record there is likely to be bias introduced by missed matches. Unlike fully automated record linkage, our approach minimised false matches given the interaction with the patient whose record was being linked. However, this approach is not scalable given the rarity of demographic surveillance sites which require substantial investment to set up. Furthermore, the PIRL database required substantial resources including computers for data entry and manpower to enter data, consent patients and run linkage protocols. As such, this approach may not be feasible within routine clinical follow-up. A more practical approach could be to introduce unique patient identifiers as recommended by the World Health Organisation.”*

**4. Please review and update the data availability statement.**

The data availability statement has now been amended (lines 385-390). We hope that this is now satisfactory.

**Reviewer #1: Comments**

**This submission by Etoori and colleagues aims to characterize undocumented transfers between health facilities, among patients who are initially classified as “lost to follow up” (LTFU), in the context of an antiretroviral treatment programme in rural South Africa. This is a useful addition to the literature, as there have been relatively few studies that have been able to trace patients who are LTFU in ART programmes, despite the high proportion of ART patients who are classified as LTFU in many African countries. A novel feature of this study is that it relies on existing population-level demographic surveillance activities to support this tracing. An important finding is that 18% of what is classified as LTFU is actually not LTFU but rather incorrectly processed records. Another important finding is that for those patients who are truly undocumented transfers, there is typically a long delay between when the patient is last seen at the original clinic and when they are seen at their second clinic. These delays are especially long for men, and this helps to understand the generally poorer levels of male engagement in HIV care in many sub-Saharan African ART programmes. The paper is mostly clear and well-written, although there are some gaps and some aspects that could be better explained.**

We thank the reviewer for their supportive comments and for highlighting the importance of our findings.

**Major comments:**

**When I first read the results, I was confused by the inconsistency between the 1017 total LTFU (reported in line 190) and the 836 LTFU in Table 1. It was only when I got to the first paragraph of the discussion that I realized the reason for the inconsistency was that the former included patients who were not ‘true’ LTFU (i.e. they had been incorrectly classified as LTFU because their transfers had actually been documented). This should have been made clearer earlier on.**

Thank you for this suggestion. We mention that “*All documented transfers are excluded from further analyses”* on line 222, and also have added “*After excluding 181 (17.8%) documented transfers, the remaining 836 patients were considered LTFU*.” at line 224-225.

**It would also be helpful if the authors could comment on why patients were incorrectly classified as LTFU. How general is this problem, and what can be done to limit this?**

In response to your suggestion, we have now added text in our discussion highlighting how data errors result in misclassification (lines 263-269).

*“High rates of misclassification of LTFU most likely reflect data errors where transfers recorded in patient files were not expeditiously updated in TIER.Net at health facilities. This misclassification also likely reflects errors in the PIRL database where transfers were not consistently captured. As TIER.Net is still not fully adopted, fully integrating it into patient care could improve data completeness and reduce these errors. Additionally, more work needs to be done to improve recording of transfers in the PIRL database.”*

**It would be good to report in the Results section how many of the undocumented transfers were identified through different means (e.g. linking to TIER versus contact through the HDSS).**

Thank you for this helpful suggestion. We have now included this information (lines 225-227).

*“Of these, undocumented transfers accounted for 134 (16.0%) of outcomes. with 63 (47.0%) identified through record review, 57 (42.5%) through PIRL, and 14 (10.5%) through supplementary tracing.”*

**It would also be good to report the overall LTFU rate, since the systematic review that the authors cite [16] shows a strong association between the LTFU rate and the proportion of LTFU cases that are undocumented transfers.**

We have now added more information on the LTFU rate (line 194)

**In Table 1 the authors report an “effect of time since last appointment”. It was unclear to me how they handled this in their statistical model, because in a Cox proportional hazards model the time variable is already implicit in the baseline hazard and you aren’t supposed to include it as a covariate. Perhaps I’m not understanding what the authors mean by “time since last appointment”, but it would seem as if they’re using the same variable as an outcome and as a covariate.**

Thank you for this helpful clarification. We have removed time since last appointment from our analyses (Table 1; line 605-606)

**Minor comments**

**Line 71 is awkwardly phrased. Silent transfer could be explained more clearly.**

Thank you for this suggestion, we have now changed the wording to make this clearer (lines 70-72)

**Lines 85-88: I found the discussion here a bit unclear and confusing. What do you mean by “migration selection effect” and when you say “increased participation” and “increased migration”, what is that increase relative to? Relative to HIV-negative individuals, relative to HIV-positive individuals in the pre-ART era, or relative to something else?**

We have edited this to make it clearer (lines 85-87)

**Line 137: I don’t think the authors previously explained what TIER.Net is. It would help if this could be explained for the benefit of non-South African readers.**

Accompanied by a reference, we provide a description of TIER.Net on lines 135-136 “*TIER.Net (the South African national HIV treatment electronic database(45)*”

**Figure 2: I don’t understand what the inner segment of the circle represents. The authors say in the footnote that it represents movements out of the facility, but it doesn’t equal the sum of the flows out of the segment.**

Thank-you for highlighting this. In order to make the figure more legible, we only visualise the largest movements and so some of the smaller flows do not appear in the figure. We apologise for causing confusion here. We have now made this clearer in the manuscript (lines 186-188, 625-638)

**Although I don’t feel strongly about it, I would consider dropping Figure 3. I don’t think it adds much to the paper, and because it doesn’t control for differences in duration of follow-up between covariate categories, it is potentially misleading. The more complete and correct analysis is already in Table 1, so presenting a simplistic analysis after you’ve already presented Table 1 is a bit distracting.**

Thank you for this suggestion. We have now dropped Figure 3 from the manuscript (lines 232, 640-643).

**Lines 224-227: I don’t understand this sentence. Why are these “time out of care” numbers so much lower than those referred to in the previous sentence?**

We apologise for this error, these numbers included both documented and undocumented transfers and were a remnant of an earlier version of the paper. We have now removed these numbers from our results (lines 242-245)

**Lines 271-3: I don’t understand why the negative association reflects “clinic shopping”. This could be explained more clearly.**

The weak negative correlation would suggest that distance to facilities may not be the only factor affecting their transfers as we would expect a much stronger correlation if this was the case. In response to your comment, we have now made this clearer in our amendments (lines 297-304)

**Lines 317-9: This sentence seems to be extrapolating too much from the data, and to put it as a final conclusion seems a bit weak. We don’t know for certain that these people were migrating because of employment, nor does this paper quantify the migration of people with HIV to ART services in the HDSS (i.e. migration in the opposite direction). If you’re only focusing on migration in one direction, you can’t really draw conclusions about how net migration flows have changed.**

We do not mean to make any inferences about net migration. However, previous research has shown that most migration in this area of South Africa, particularly to economic hubs, is connected with labour migration. Given that these are known residents of the HDSS, we think it would be reasonable to assume that movement to economic hubs might be linked to labour migration especially given South Africa’s particular history. We have amended the statement to reflect some uncertainty in this finding (line 360).

**Reviewer #2:**

**The manuscript provides a thorough analysis of documented and undocumented clinic transfers among PLWH considered lost to follow-up after ART initiation. While the concept is not novel, the authors add some new information.**

**My biggest problem with the manuscript is that in at least 2 places it copies nearly directly from an earlier published article -- listed as #6 on the reference list -- both in descriptions of the Methods, and also in the Discussion. For example, from the original article (published in JAIDS in 2017), "Manually searching for lost patients took many hours and is more feasible as a research exercise than as a routine clinical follow-up." The submitted manuscript states on lines 302-304: "Furthermore, searching for patient outcomes involved time consuming manual procedures and is more feasible as a research exercise than within clinical follow-up." That statement is nearly verbatim to the original statement and is too similar to be published in the manner presented, especially with no citation.**

I am regretful for this oversight. In writing the manuscript, ideas were copied from the published article you mention in order to guide my own writing but in the editing process, I lost track of these and did not rephrase them or cite them properly in the manuscript I submitted. I apologise unreservedly for this error. We have now rephrased or deleted statements that are similar to this publication and have also added citations to credit the authors (lines 172-176, 308-310, 332-333).

**Beyond that troubling aspect, a major problem with the paper is that it does not account for required transfers during pregnancy. In South Africa, maternity care typically occurs at Midwife Obstetrics Units (MOUs) that are separate from ART facilities. After delivery, women receiving care at MOUs typically face mandatory transfer back to the ART unit for routine HIV care in the postpartum period. This was well described in citation number 33 by TK Phillips, et al, in JIAS (2018). However, the authors of the submitted manuscript do not describe the transfer requirements in place at the study sites, even though it may influence the outcome of the study, since movement among pregnant/postpartum women was found to be high.**

Thank you for this helpful comment. We have now added this important information to the manuscript (lines 110-111, 282-284)

*“In this setting, pregnant women must transfer their HIV care from integrated HIV and antenatal care facilities to general ART facilities 6-10 weeks after delivery.”*

*“Furthermore, postpartum women in South Africa are expected to transfer their treatment to general ART clinics after delivery which might further drive these movements.”*

**In general, the Results are not presented very clearly. For example, lines 243-245 of the Discussion state an important point: 17.8% of LTFU were in error. But this finding is muddled in the Results. When I tried to find where the 836 number came from (line 212), I had to pull out my calculator myself. Similarly, the authors present Figures 2-5, which are fairly sophisticated from an analysis standpoint but are barely mentioned in the Results, let alone described and explained.**

Thank you for this suggestion. We mention that documented transfers are excluded from further analysis on line 222, and have now made this more explicit by presenting the pre and post excluded numbers (line 224-225)

We have also described the Figures within the text (lines 211-214, 220-222, 235-238).

We have further clarified how to interpret Figures 2 and 4 (lines 625-638)

**Lines 70-72 include a sentence that is awkward and needs revision.**

Thank you for this suggestion, we have now revised this sentence (lines 70-72)

**Lines 187-188 note that 737 met the ART initiation criteria for non-pregnant adults. What were these criteria at the time of data collection? Define in the Methods.**

Thank you for this suggestion, we have now added this to the Methods section (lines 109-110).

*“At the time of this study, South Africa has moved to the universal test and treat strategy.”*

**Table 1: Do the 2 columns for HR and p-value represent crude and adjusted values? Something else? An important header is missing.**

We have added a footnote to the table to make this clearer (lines 607)
